# Caloric restriction controls stationary phase survival through Protein Kinase A (PKA) and cytosolic pH

**DOI:** 10.1111/acel.12921

**Published:** 2019-02-20

**Authors:** Laura Dolz‐Edo, Margaretha van der Deen, Stanley Brul, Gertien Jacoba Smits

**Affiliations:** ^1^ Department of Molecular Biology and Microbial Food Safety Swammerdam Institute for Life Sciences, University of Amsterdam Amsterdam The Netherlands

**Keywords:** calorie restriction, cAMP, chronological lifespan, glucose, intracellular pH, *Saccharomyces cerevisiae*

## Abstract

Calorie restriction is the only physiological intervention that extends lifespan throughout all kingdoms of life. In the budding yeast *Saccharomyces cerevisiae,* cytosolic pH (pH_c_) controls growth and responds to nutrient availability, decreasing upon glucose depletion. We investigated the interactions between glucose availability, pH_c_ and the central nutrient signalling cAMP‐Protein Kinase A (PKA) pathway. Glucose abundance during the growth phase enhanced acidification upon glucose depletion, via modulation of PKA activity. This actively controlled reduction in starvation pH_c_ correlated with reduced stationary phase survival. Whereas changes in PKA activity affected both acidification and survival, targeted manipulation of starvation pH_c_ showed that cytosolic acidification was downstream of PKA and the causal agent of the reduced chronological lifespan. Thus, caloric restriction controls stationary phase survival through PKA and cytosolic pH.

## 1 INTRODUCTION

Reduction of calorie intake, also known as calorie restriction (CR), is the only physiological intervention that universally extends lifespan. In budding yeast, we can distinguish two separate but related aspects of cellular lifespan: Replicative lifespan refers to the number of times a cell can divide and provides a model for the aging process of mitotic or stem cells in metazoans. Chronological lifespan (CLS) refers to the time a non‐dividing cell can survive, corresponding to postmitotic longevity. In yeast, CLS is usually assessed in stationary phase cultures, when all carbon sources have been exhausted. CR, defined as a reduction of glucose levels in the media (from 2% to 0.5% glucose) extends both aspects of lifespan (Lin, 2000; Murakami, Burtner, Kennedy, & Kaeberlein, 2008).

The balance between cell growth and survival in response to both extracellular and intracellular stimuli is coordinated by signalling pathways, in order to adapt to changing environments (Broach, 2012; Ho & Gasch, 2015). One of the key signalling nodes in *Saccharomyces cerevisiae* is the Protein Kinase A (PKA) pathway. The PKA pathway is essential for growth and responds primarily to glucose and other fermentable sugars (Conrad et al., 2014). While stimulating growth, PKA signalling suppresses stress responses (Conrad et al., 2014). PKA has a prominent role in transitions of carbon availability. PKA activation is necessary for the transcriptional reprogramming occurring upon glucose addition to cells growing on poor carbon sources (Zaman, Lippman, Schneper, Slonim, & Broach, 2009). Indeed, direct artificial activation of the pathway is sufficient to recapitulate most of the glucose‐dependent transcriptional response observed in such cultures. Proper PKA inactivation is also required for survival during nutrient‐poor conditions. When cultures are subjected to severe carbon starvation during stationary phase, over‐activation of the PKA pathway shortens CLS, while mutations that reduce its activity are well known to extend viability (Fabrizio et al., 2003).

The main regulation of PKA kinase activity is by fermentable sugars, and consequently, most research has focused on elucidating the glucose signalling mechanism. The PKA kinase is a heterotetramer composed of two regulatory (Bcy1) and two catalytic subunits (Tpks) in its inactive form. Activation of the kinase occurs when the second messenger cAMP binds to the regulatory subunits, releasing the catalytic subunits, which are encoded by three partially redundant isoenzymes (Conrad et al., 2014; Thevelein & De Winde, 1999). Therefore, cAMP levels are key for PKA regulation. Glucose addition to de‐repressed cultures induces a transient cAMP increase by the activation of adenylate cyclase (Cyr1) via two branches of the pathway: Ras and the G protein‐coupled receptor system. Of these two branches, only Ras signalling is essential for PKA activation and growth (Conrad et al., 2014). The concentration of cAMP is downregulated via degradation by the phosphodiesterases Pde1 and Pde2 (Ma, Wera, Dijck, & Thevelein, 1999). While the phosphodiesterases and other regulators of [cAMP] are upstream of PKA, they are PKA targets themselves, contributing to a negative feedback mechanism and the transient nature of the glucose‐induced cAMP peak (Vandamme, Castermans, & Thevelein, 2012).

PKA inactivation at diauxic shift is required for proper diauxic transition, post‐diauxic growth and stationary phase survival or CLS (Boy‐Marcotte et al., 1996; Russell, Bradshaw‐Rouse, Markwardt, & Heideman, 1993). However, very little is known about the mechanisms for PKA inactivation when glucose becomes depleted at the diauxic shift. The levels of the inhibitory Bcy1 increase around this time, which was assumed to contribute to PKA inhibition (Winderickx et al., 2003). However, Tpk1 and Tpk2 levels increase in parallel to Bcy1 and PKA may therefore not be inhibited by this additional cAMP/Bcy1 control (Tudisca et al., 2010). Whether changes in the localisation of the Tpks and Bcy1 upon glucose depletion contribute to the inhibition, remains to be stablished (Tudisca et al., 2010).

Changes in cytosolic pH (pH_c_) alter the protonation state ratio of all weak acid and basic groups present in the cytosol, thereby potentially affecting most if not all processes occurring inside a cell (Orij, Brul, & Smits, 2011). Recently pH_c_ has been shown to function as a second messenger regulating gene expression (Young et al., 2010), G protein‐mediated signalling (Isom et al., 2013), growth (Dechant, Saad, Ibáñez, & Peter, 2014; Orij et al., 2012) and aging (Henderson, Hughes, & Gottschling, 2014) in yeast. In higher organisms, intracellular pH appears to have similar roles and its dysregulation has been linked to cancer progression and neurodegenerative diseases (Harguindey et al., 2017; White, Grillo‐Hill, & Barber, 2017).

It is therefore interesting to note that pH_c_ is strongly influenced by nutrient availability. Whereas the pH in the cytosol remains around neutral values during growth on glucose, upon glucose depletion at the end of the growth phase, pH_c_ decreases ~1 pH unit (Orij et al., 2012). Imposed abrupt glucose starvation also leads to a strong decrease of pH_c _(Dechant et al., 2010). A small pH_c_ decrease during the normal growth phase has been shown to act as a growth limiting signal. The signal transduction of this control remains unclear (Orij et al., 2012), but an interaction with regular nutrient signalling is to be expected.

Intracellular pH was proposed to control PKA, but different and apparently opposite modes of control have been reported. Intracellular acidification by addition of protonophores at low pH is able to trigger cAMP induction and concomitant activation of PKA, similar to how glucose addition to de‐repressed cells activates the pathway (Thevelein & De Winde, 1999). Low intracellular pH triggers cAMP synthesis via Ras (Colombo et al., 1998), but also via direct biochemical regulation, as adenylate cyclase activity increases at acidic pH (Purwin, Nicolay, Scheffers, & Holzer, 1986). Glucose addition to starved cells itself *causes* a transient cytosolic acidification, in a timescale similar to the cAMP peak (Tarsio, Zheng, Smardon, Martínez‐Muñoz, & Kane, 2011). Whether it actually is the glucose‐dependent transient acidification that triggers cAMP induction upon glucose re‐addition remains unclear; the two have been suggested to be independent, although kinetic analyses reveal that the pH_c_ decrease precedes the cAMP response (Thevelein et al., 1987).

In contrast to the previous findings, low pH_c_ has been proposed to *inactivate* PKA via regulation of the vacuolar H^+^‐ATPase (V‐ATPase). Disassembly of V‐ATPase responds to pH_c_ perturbations and lack of V‐ATPase decreases PKA activity both upon glucose depletion and re‐addition (Dechant et al., 2010, 2014). Acidification by Pma1 depletion also caused Ras inhibition and a pH‐dependent growth arrest (Dechant et al., 2014). Overall, these data support a model in which cytosolic acidification inhibits PKA activity, at least partially via Ras, which appears to contradict the observations that showed a *positive* effect of acidification on cAMP/PKA activity.

In addition to pH control of PKA, PKA was found to regulate both plasma membrane and vacuolar H^+^‐pumps, Pma1 and V‐ATPase (Bond & Forgac, 2008; Souza, Trópia, & Brandão, 2001), which suggests a role of PKA in pH_c_ control. Here, we address this question and show that pH_c_ is controlled by PKA in a glucose concentration‐dependent manner and that these changes in pH_c_ are an important determinant of calorie restriction control of CLS.

## 2 RESULTS

### 2.1 PKA promotes cytosolic acidification upon glucose depletion

**Figure 1:**
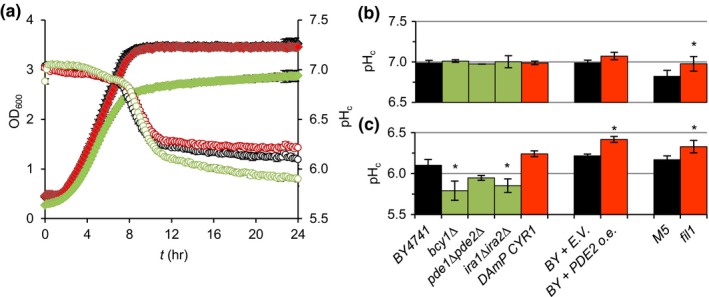
Genetic manipulation of PKA activity modulates pH_c_ upon glucose depletion. Mutants with overactive PKA activity (green) have reduced pH_c_ and low PKA activity mutants (red) an increased pH_c_ when glucose is depleted from the media. (a) BY4741 (black), *bcy1Δ *(green) and DAmP *CYR1* (red) strains were grown in microplates and OD_600_ (filled symbols) and pH_c_ (open symbols) were monitored. A representative example is shown. (b) and (c) summarise pH_c_ during exponential growth (b) and after glucose depletion (c) for the set of PKA mutants analysed (See Materials and Methods for details). Representative OD_600_ and pH_c_ curves for each strain can be found in Supporting information Figure S1. Data represent average ± standard deviation (*SD*) of at least three biological replicates per strain

To study how the PKA pathway is involved in the regulation of pH_c_, we monitored pH_c_ and OD_600_ (optical density at 600 nm) over the course of the progression through all growth phases in a set of mutants previously described to either over‐activate or reduce the activity of the pathway. PKA over‐activation was achieved by deletion of the PKA regulatory subunit (*bcy1Δ*), deletion of the phosphodiesterases (*pde1Δpde2Δ*) and deletion of the Ras regulatory proteins (*ira1Δira2Δ*). Decreased PKA activity was induced by destabilisation of adenylate cyclase mRNA (*DAmP CYR1*), overexpression of one of the phosphodiesterases (*PDE2 o.e.*) and expression of an adenylate cyclase allele with reduced activity (*fil1*). As previously described (Orij et al., 2012), the wild‐types maintained pH_c_ around neutrality during exponential growth, while pH_c_ decreased almost 1 unit when glucose was depleted. Qualitatively, the PKA mutants analysed presented a pH_c_‐time profile similar to their respective parental strain, with a pH_c_ around seven during growth and a strong acidification upon glucose depletion (Figure 1a, Supporting information Figure S1). Systematic quantitative assessment of pH_c_ during the exponential growth phase (Figure 1b) and a set time after glucose depletion (Figure 1c) revealed that altered PKA activity did not strongly affect pH_c_ during exponential growth; only the low PKA activity mutant *fil1*, presented a pH_c_ significantly different from its parental strain. Upon glucose depletion, however, we did observe a clear effect of PKA pathway mutations on pH_c_ (Figure 1c). The strains with overactive PKA, *bcy1Δ* and *ira1Δira2Δ,* had a significantly lower pH_c_ than the parental strain whereas the strains with downregulated PKA, *fil1 *and *PDE2 o.e.,* had a significantly higher pH_c_ than their respective parental strains 10–11 hr after glucose depletion. The strains *pde1Δpde2Δ* and *DAmP CYR1*, with respectively overactive and reduced PKA activity fit within this trend, but the difference was not significant. These results indicate that the PKA pathway affects pH_c_ specifically when glucose is depleted.

**Figure 2:**
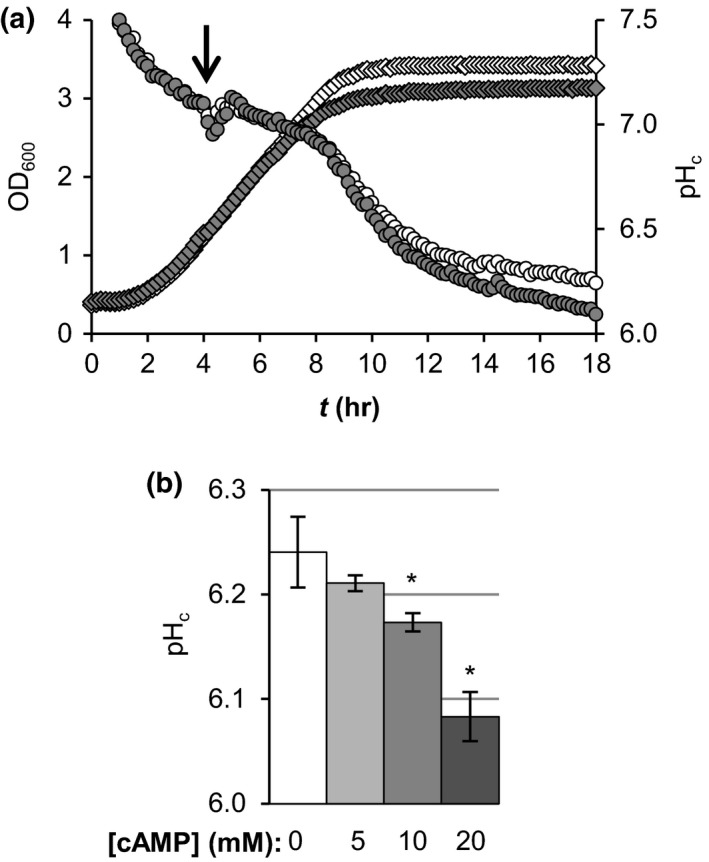
Addition of cAMP during exponential growth promotes cytosolic acidification upon subsequent glucose depletion. (a) OD_600_ (diamonds) and pH_c_ (circles) were monitored during growth of the parental strain BY4741. 20 mM cAMP (closed symbols) or water (open symbols) were added to the cultures after 4 hr of growth (arrow). A representative example is shown. (b) Comparison of the pH_c_ at the end of the growth curve (18 hr) for the cAMP treatments indicated. Data are averages ±*SD* of three biological replicates

Direct activation of PKA with cAMP in wild‐type cells also affected pH_c_ upon glucose depletion. We treated BY4741 exponentially growing cultures with cAMP and monitored OD_600_ and pH_c_ before and after the treatment (Figure 2a). During growth in the presence of glucose, cultures treated with cAMP did not have an altered pH_c_, but upon glucose depletion they showed a stronger cytosolic acidification in a dose‐responsive manner (Figure 2b). Addition of cAMP during growth in the strain lacking the phosphodiesterases (*pde1Δpde2Δ*) caused a stronger effect on pH_c_ upon glucose depletion than in the parental strain, as expected due to reduced cAMP degradation (Supporting information Figure S2). This further supported the idea that PKA activity promotes cytosolic acidification when glucose is depleted.

**Figure 3:**
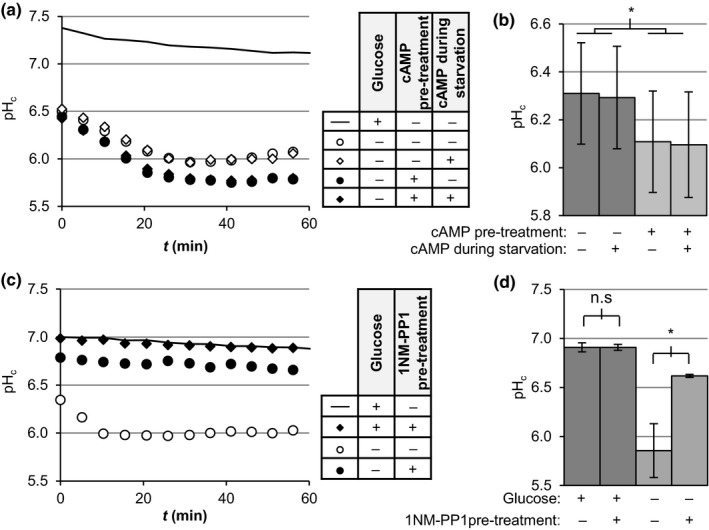
Manipulation of PKA activity before glucose depletion modulates pH_c_ upon glucose starvation. (a, c) Exponentially growing BY4741 (a) or *TPK1^as^tpk2∆tpk3∆* (c) cultures were subjected to the treatments indicated in the adjacent table and transferred to a microplate to monitor pH_c_ as described in Materials and Methods. Data show a representative result. Pre‐treatments indicate incubation for 90 min prior to starvation with 20 mM cAMP (a) or 2 µM 1NM‐PP1 (c), while cAMP during starvation in (a) indicates the cells were washed with and starved in media without glucose containing 20 mM cAMP. (b, d) pH_c_ 60 min after starvation for the indicated treatments as in (a) or (c), respectively. Data show averages ±*SD* of three biological replicates. Significance was tested using two‐way ANOVA with matching for both factors and Bonferroni's multiple comparisons test

To test the effect of PKA on pH_c_ independently of growth history, including medium composition (i.e. nutrients available or growth by‐products), we performed controlled glucose starvation experiments, in which we washed and resuspended cultures in medium without glucose with various cAMP treatment regimens (Figure 3a–b). In agreement with our previous observations, cAMP addition or pre‐treatment had no effects on pH_c_ in the presence of glucose (Supporting information Figure S3). Resuspension of the cells in medium without glucose decreased pH_c_ (Figure 3a, open circles vs. line), mimicking the effects of glucose depletion observed in growth curves. Surprisingly, addition of cAMP during starvation did not induce additional changes in pH_c_ (Figure 3a, open diamonds vs. open circles). However, when we treated with cAMP for 90 min and then starved the cells for glucose, we observed enhanced acidification (Figure 3a, closed vs. open circles), similar to the effect observed in mutants with overactive PKA. This was independent from the presence of cAMP during the starvation period (Figure 3b), indicating that induction of PKA in the *presence* of glucose promotes acidification upon glucose starvation and suggesting that PKA activity during (the late phases of) growth determines pH_c_ upon glucose depletion.

Protein Kinase A is essential for growth and genetic manipulations that completely inactivate the pathway are lethal, unless compensated with additional mutations (Broach, 2012). An alternative way to inhibit PKA is the use of strains carrying ATP analogue‐sensitive (as) mutations. These point mutations do not affect kinase activity under control conditions but render the catalytic subunit sensitive to the ATP analogue 1NM‐PP1 and allow inhibition of the kinase activity by addition of the drug to the media (Stephan, Yeh, Ramachandran, Deminoff, & Herman, 2009; Zaman et al., 2009). In order to evaluate the effect of PKA inhibition on pH_c_, we performed starvation experiments with the strain *TPK1^as^tpk2∆tpk3∆*, which carries a single analogue‐sensitive catalytic subunit of PKA and allows inactivation of PKA kinase activity at any time by 1NM‐PP1 addition. Inactivation of the kinase was induced by incubating the cultures with 2 µM of 1NM‐PP1 before the starvation as previously (Aoh, Graves, & Duncan, 2011). This concentration inhibited growth in cultures of *TPK1^as^tpk2∆tpk3∆* but not in the parental strain (Supporting information Figure S4; and Stephan et al., 2009), suggesting that PKA was specifically inhibited in this mutant. Addition of 1NM‐PP1 to wild‐type cultures did not affect the pH_c_ in the presence of glucose and did not have additional effects on the acidification upon glucose starvation (Supporting information Figure S5). As with mutants inactivating PKA, and opposite to PKA activation with cAMP, treatment of the *TPK1^as^tpk2*∆*tpk3*∆ cells with 1NM‐PP1 had no effect on pH_c_ in the presence of glucose, but pre‐treatment with the inhibitor delayed and significantly reduced the extent of acidification upon glucose starvation (Figure 3c–d). These effects are independent of strain background (Supporting information Figure S6).

PKA promoted pH_c_ decrease after gradual glucose consumption at the end of the growth phase (glucose depletion; Figures 1 and 2), as well as when glucose was removed from growing cultures (glucose starvation; Figure 3), indicating that the mechanism behind the regulation of pH_c_ upon gradual (glucose depletion) versus sudden glucose removal (starvation) is the same. Taken together, our results show that PKA activity, set when glucose is still present, regulates pH_c_ upon glucose starvation. Induction of the PKA pathway in the presence of glucose enhances cytosolic acidification during glucose starvation, whereas a decrease of PKA activity strongly reduces the glucose starvation‐induced pH_c_ decrease.

### 2.2 Calorie restriction controls pH_c _via PKA

**Figure 4:**
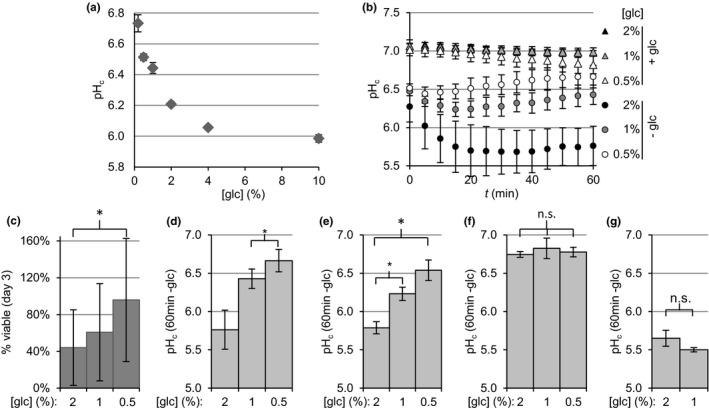
The initial concentration of glucose in the media regulates starvation pH_c_ via PKA. (a) pH_c_ after glucose depletion (calculated as in Figure 1c) of BY4741 cultures after growth at the indicated initial concentrations of glucose (glc). A representative growth and pH_c_ curve can be found in Supporting information Figure S7. (b) pH_c_ dynamics of BY4741 cultures starved for glucose after growth at the indicated initial concentrations of glucose (c) Cell viability after three days of glucose starvation as in panel B. (d–g) Cytosolic pH 60 min after glucose starvation for BY4741 (d), *TPK1as tpk2Δ tpk3Δ* without (e) or with (f) 1NM‐PP1 pre‐treatment as in Figure 3c–d, and *bcy1Δ* (g). Data shown are averages ±*SD* of three biological replicates. Significance was tested using one‐way ANOVA with matching with Bonferroni's correction

The fact that PKA activity *before* glucose depletion sets pH_c_ upon glucose starvation reminded us of CR effects on CLS, where glucose levels during growth affect viability after glucose depletion (Murakami et al., 2008). We asked whether CR would also affect starvation pH_c_, similarly to PKA manipulation. We inoculated yeast in a range of glucose concentrations and monitored OD_600_ and pH_c_ during lag phase, growth and after glucose depletion (Supporting information FigureS7), observing a dose‐dependent decrease of pH_c_ after glucose depletion as glucose concentration increased (Figure 4a). In controlled starvation experiments, we grew cultures on media containing 2% (control) as well as 1% and 0.5% glucose (CR), starved them for glucose and monitored pH_c_ as well as viability 3 days later. Decreasing glucose levels did not influence pH_c_ in the presence of glucose, but significantly reduced cytosolic acidification upon starvation (Figure 4b, d) which correlated with increased viability three days later (Figure 4c).

To confirm that the glucose control of pH_c_ is mediated by PKA, we reasoned that the manipulation of PKA activity should abolish the effects of glucose concentration on pH_c_. We inhibited PKA using the *TPK1^as^tpk2∆tpk3∆ *mutant, and found that, indeed, starvation pH_c_ now remained high, and became insensitive to the glucose concentration (Figure 4e–f). Complementarily, we analysed starvation pH_c_ in the mutant *bcy1Δ* which lacks the regulatory subunit of the kinase and therefore has fully active PKA. Cultures lacking *BCY1* presented a similarly low starvation pH_c_ after growth at both 2 and 1% glucose (Figure 4g). We do not show data for 0.5% glucose because *bcy1Δ* cultures had depleted this low amount of glucose within the time of the experiment (see Experimental Procedures). Therefore, glucose availability during growth and prior glucose depletion regulates starvation pH_c _via PKA.

### 2.3 Effects of cytosolic pH on starvation survival

**Figure 5:**
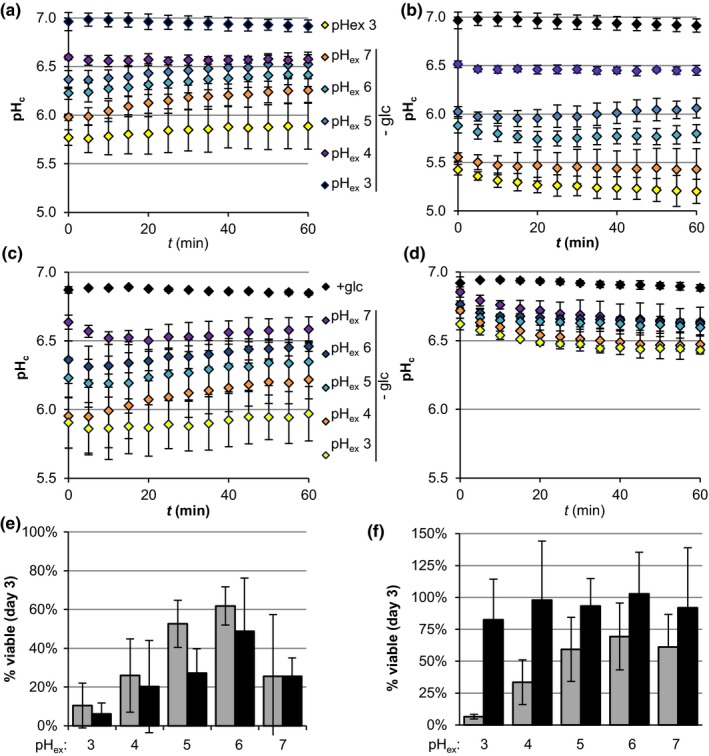
Cytosolic acidification correlates with viability upon glucose starvation. (a, b) pH_c_ dynamics of BY4741 cultures starved for glucose (glc) at different extracellular pH (pH_ex_) in the absence (a; DMSO) or presence (b) of ebselen. (c, d) pH_c_ dynamics of *TPK1^as^tpk2∆tpk3∆ *cultures starved for glucose at different pH_ex_ after a control treatment with DMSO (c) or a treatment with 0.2 µM of 1NM‐PP1 (d) for 90 min prior to starvation. (e, f) Cell viability after three days of glucose starvation for (e) BY4741 cultures as in a and b or (f) for *TPK1^as^tpk2∆tpk3∆ *cultures as in c and d. Grey bars represent control treatments. Black bars represent ebselen treatment (e) or 1NM‐PP1 pre‐treatment (f). Data shown are averages ±*SD* of three biological replicates

The fact that PKA actively controls pH_c_ during starvation suggests a functional role of pH_c_ in the adaptation to non‐glucose conditions. To test this hypothesis, we studied the consequences of changes in starvation pH_c_ on viability during starvation. Cytosolic pH in the absence of glucose depends on the pH of the medium (extracellular pH; pH_ex_); Therefore, we manipulated starvation pH_c_ by starving cultures for glucose in media with a pH_ex_ in a range from 3–7 and determined viability three days after starvation by colony‐forming units counts. Cytosolic pH during glucose starvation decreased with pH_ex_ (Figure 5a). Between pH_ex_ 3–6, starvation survival correlated strongly with pH_c_, with viability decreasing at lower pH_c_ (Figure 5e, grey bars); at pH_ex_ 7, this correlation collapsed. The pH‐dependent viability profile was also maintained at later times (Supporting information Figure S8A) and in CLS experiments (Supporting information Figure S8C), suggesting pH could be a factor regulating stationary phase survival. The low viability observed at both extremes of the pH range could be due to either *extracellular* pH or *cytosolic* pH. In order to distinguish between these options, we manipulated pH_c_ in a pH_ex_‐independent way. We imposed an additional decrease on pH_c_ in the absence of glucose by inhibiting the plasma membrane H^+^‐ATPase Pma1 with ebselen (Young et al., 2010) and evaluated its consequences on viability. If pH_c_ was the cause of reduced viability at both low and high pH_ex_, further decreasing pH_c_ with ebselen should reduce viability at all pH_ex_ except at pH_ex_ 7. Ebselen treatment decreased pH_c_ at all starvation pH_ex_ values (Figure 5a‐b) and this reduced viability in all conditions except for pH_ex_ 7 (Figure 5e, black vs. grey bars), consistent with the hypothesis that acidification of the cytosol decreases starvation survival.

Our data suggest that acidification of the cytosol upon glucose depletion reduces CLS. If this is a direct causal relationship, increasing pH_c_ during starvation should improve starvation survival. We attempted to increase pH_c_ by inducing *PMA1* overexpression prior the starvation (Henderson et al., 2014), but found no significant effects on starvation pH_c_ (Supporting information Figure S9). Therefore, to induce a high starvation pH_c_, we manipulated PKA activity in the *TPK1^as^tpk2∆tpk3∆ *strain, using as low a dosage of 1NM‐PP1 as possible, which still induced a significant increase in pH_c_. Starvation of untreated *TPK1^as^tpk2∆tpk3∆ *cultures at a range of pH_ex_ leads to a pH_c_ response similar to wild‐type (Figure 5c). Inhibition of PKA activity prior the starvation prevented the decrease of pH_c_ (Figure 5d), as expected from our previous observations. Under these conditions, the starvation pH_c_ became mostly insensitive to pH_ex _and stabilized around 6.5 in all starvation conditions. The survival three days after starvation for control *TPK1^as^tpk2∆tpk3∆ *was again similar to wild‐type (Figure 5f, grey bars). As hypothesised, 1NM‐PP1 treatment abolished not only the pH_c_ response to pH_ex_ but also the loss of viability (Figure 5f, black bars), supporting the idea that the PKA‐controlled reduction in pH_c_ limits starvation viability.

## 3 DISCUSSION

### 3.1 PKA control of starvation pH_c_

The Protein Kinase A (PKA) pathway is a key regulator of cellular responses, coordinating the balance between growth and stress responses. Recently, pH_c_ has also been shown to regulate growth (Dechant et al., 2014; Orij et al., 2012). More specifically, pH_c_ seems to be a sensor of environmental or metabolic state, connecting carbon source availability with growth regulation. PKA and pH_c_ share a common major input (carbon source availability) and output (growth control). Hence, they presumably interact to ensure a coordinated response. Literature addressed part of this interaction and reported effects of pH_c_ on PKA with conflicting results (Colombo et al., 1998; Dechant et al., 2014). However, the complementary possibility, PKA control of pH_c_, had not been addressed so far.

We analysed the effect of manipulation of PKA activity on pH_c_ (Figures 1 and 2). PKA activity did not regulate pH_c_ during growth, but we did observe a strong role of PKA in pH_c_ control upon glucose depletion, which was recently corroborated (Isom et al., 2018). This is remarkable, for two reasons. First, it was usually assumed that starvation acidification was passive, because of the absence of energy and consequently the inactivation of the ATPase Pma1. But we found that Pma1 inhibitor ebselen could further reduce starvation pH_c_, a clear indication of activity of the pump (Figure 5a–b). The fact that a PKA inactivating mutation resulted in a higher starvation pH_c, _independently of pH_ex _(Figure 5d), also shows that starvation pH_c_ is an actively controlled property. Secondly, activity of PKA itself requires cAMP and ATP, both of which are low after glucose depletion (Ashe, Long, & Sachs, 2000; Russell et al., 1993), and the pathway is thought to inactivate upon diauxic shift (Thevelein & De Winde, 1999). Careful assessment of the effect of timing of the cAMP addition showed that it was the activity of PKA as set *prior to* glucose starvation and not during the starvation itself that controlled the starvation pH_c_ (Figure 3a–b). Together, these results show that the level of PKA activity, controlled by glucose abundance before glucose depletion when the pathway is still active, actively controls pH_c_ in the absence of glucose. A similar role for PKA activity *during* growth controlling processes *after* glucose depletion was observed in the delocalisation of trans‐Golgi/endosomal adaptors upon acute glucose depletion (Aoh et al., 2011), suggesting a common mechanism for PKA control in the glucose to non‐glucose transition. The fact that different initial concentrations of glucose (Figure 4) or addition of cAMP (Figures 2 and 3) quantitatively set pH_c_ via PKA modulation also shows that PKA is not a mere on/off switch but that it is quantitatively regulated.

How PKA regulates pH_c_ is not known but the main pH_c_ regulators, the H^+^‐ATPase's Pma1 and V‐ATPase are the most likely candidates. PKA inhibition increases ATP levels upon glucose depletion (Aoh, Hung, & Duncan, 2013), which is required for proton pumping and thus may explain the increased starvation pH_c_ when PKA is inactivated. On the other hand, a reduced need for proton pumping may be the cause of the observed ATP conservation. Besides substrate control of activity, both vacuolar and plasma membrane pumps undergo post‐translational modifications in response to glucose transitions. Pma1 is found inactive and dephosphorylated upon glucose starvation, and glucose addition leads to Pma1 phosphorylation, but neither activating kinase(s) nor inactivating phosphatase(s) have been identified (Kane, 2016). Inactivation of V‐ATPase upon starvation occurs via reversible disassembly of the *V*_1_ and *V*_0_ subunits and seems pH_c_‐dependent (Dechant et al., 2010). This dissociation was impaired in PKA overactive mutants (Bond & Forgac, 2008), although others suggested that PKA is downstream of V‐ATPase instead (Dechant et al., 2010, 2014).

### 3.2 The importance of pH_c_ in starvation survival

The active control of cytoplasmic acidification by PKA suggests that this low pH_c_ during starvation is beneficial. What we found, however, was a strong correlation between an acidic starvation pH_c_ and decreased viability after three days below neutral pH_ex_ (Figure 5a–b, e). Low viability at neutral/alkaline starvation pH_ex_ has been associated with lack of enzyme aggregate formation (Petrovska et al., 2014). At neutral pH_ex_, factors other than pH_c_ may contribute to the viability loss, as suggested by the lack of effect of ebselen (Figure 5e). For instance, at pH_ex_ 7, pH_c_ is lower than pH_ex_, reversing the normal proton gradient. Similar to what occurs during growth at alkaline pH_ex_, this may interfere with nutrient import, which is often coupled to H^+ ^symport (reviewed in Ariño, 2010).

It seems remarkable that pH_c_ changes so small might have such strong effects, as we understand that a change of 0.2 pH units will change the protonation state of one group by a factor of only 1.6. It should be noted, however, that pH sensing by macromolecules is already complex; several accessible or hidden amino acids together may form a pH sensing network (Isom et al., 2013), and complexity is added when considering receptor–ligand or enzyme–substrate interactions where both partners can be pH sensing. Phosphorylation, with a pK_a_ completely in the physiological range, adds another layer of sensitivity (Young et al., 2010).

In good agreement with low pH_c_ limiting starvation survival, blocking cytosolic acidification upon glucose depletion by inhibition of PKA resulted in fully retained viability upon starvation (Figure 5c–d, f). This PKA inactivation likely activated the transcription factors Msn2/4 (Görner et al., 1998), so we cannot exclude that this PKA inactivation also affected viability through Msn2/4 and the induction of stress resistance genes (Fabrizio et al., 2003).

The ultimate experiment to show that pH_c_ is the main determinant of survival downstream of PKA is to rescue the low viability of a high PKA mutant by increasing pH_c_. We attempted to increase pH_c_ in a number of ways. All our attempts at restoring starvation pH_c_ independently from PKA failed, showing all the more how robustly this reduced pH_c_ is controlled. We increased pH_ex_ in the PKA overactive mutant* ira1Δira2Δ. *This too failed to fully compensate for the strong acidification, and the pH_c_ of *ira1Δira2Δ *remained lower than wild‐type for all the tested pH_ex_ (Supporting information Figure S10A–B). The effects on viability of the small pH_c_ increase from pH_ex_ 5 to 6 were rather limited, but increased survival, although the low pH_c_ in *ira1Δira2Δ* caused a lower survival than in the parental strain at any pH_ex_ (Supporting information Figure S10C–D).

We conclude that PKA‐controlled pH_c_ is a major factor in the control of starvation survival. Yeast cultures naturally experience such severe carbon starvation conditions in stationary phase. Although the gradual transition into stationary phase likely involves additional adaptations compared to sudden starvation, several observations support the idea that a low pH_c_ regulates stationary phase survival (CLS) as well. First, growth on media at a low pH_ex_, which enhances cytosolic acidification after glucose depletion (Supporting information Figure S8B), shortens CLS too (Figure S8C and Burtner, Murakami, Kennedy, & Kaeberlein, 2009; Fabrizio et al., 2004). Second, addition of acetic acid reduces CLS only at low pH_ex_ (Burtner et al., 2009), conditions in which the acid is protonated and therefore can diffuse through the plasma membrane and acidify the cytosol (Kane, 2016). Thus, our observations together with previous literature support that *cytosolic* pH rather than acidic media reduces CLS.

**Figure 6:**
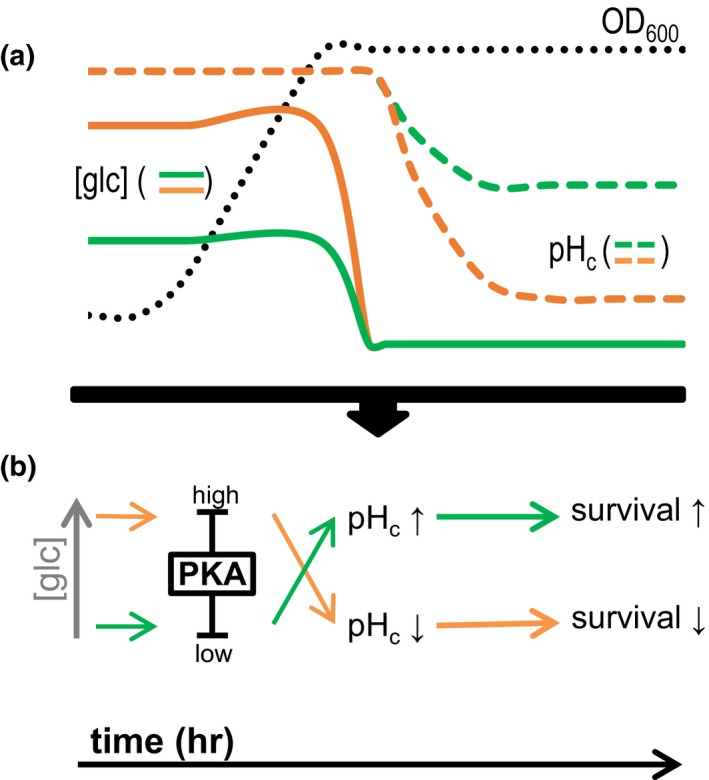
Model for the glucose‐dependent control of survival via PKA and pH_c_ proposed in this work. (a) Schematic representation of the data. Solid lines represent glucose concentrations ([glc]), and dashed lines represent pH_c_ measurements. Growth (OD_600_) is represented by a black dotted line. (b) Model derived from the data. During growth, glucose levels activate PKA quantitatively. When glucose is depleted from the media, pH_c_ decreases according to the level of PKA activity. Orange represents high glucose concentrations, which, via induction of high PKA activity, trigger a low pH_c_ upon glucose depletion and limit survival. Green represents low glucose, which leads to lower PKA activity and thus increases pH_c_ upon glucose depletion and survival

Glucose abundance promotes CLS via regulation of the PKA signalling pathway (Fabrizio et al., 2003, 2004; Murakami et al., 2008; Wei et al., 2008). Here, we show that glucose abundance promotes cytosolic acidification via PKA and that this enhanced acidification limits CLS. Thus, taken together our data support a model in which increased glucose availability, via quantitative activation of PKA, promotes the decrease of pH_c_ upon starvation, which is one of the mechanisms decreasing viability upon CLS (Figure 6).

This is remarkable: Why would yeast *actively* decrease pH_c_ if it reduces survival? An explanation for this apparent paradox would be that acidification also has a positive impact on physiology besides the detrimental aspect uncovered here. For instance, acidification‐triggered protein aggregation has been proposed to inactivate enzymes and protect them from damage, facilitating growth resumption when glucose is again available (Petrovska et al., 2014). Also, the key pH_c_ regulator Pma1 is estimated to consume around 20% of cellular ATP under normal conditions (Ambesi, Miranda, Petrov, & Slayman, 2000). Hence, inhibition of the ATP‐dependent H^+^‐pumps may reduce energy expenditure at the cost of decreasing pH_c_. An alternative is that the unfavourable pH_c_ decrease may be a consequence of the favourable PKA‐dependent fast fermentative growth programme. PKA activity may have evolved to balance between fast growth and survival, with dysregulations affecting both (Zakrzewska et al., 2011; Zaman et al., 2009). Such a growth‐versus‐survival trade‐off is similar to the model of quasi‐programmed aging, according to which the age‐related death would be an aftermath of the growth programme (Arlia‐Ciommo, Piano, Leonov, Svistkova, & Titorenko, 2014). Alternatively, the death of a fraction of the population may be beneficial too, as it releases nutrients that can be used for growth by the remaining (genetically identical) more adapted cell subpopulation (Fabrizio et al., 2004). What causes cells to lose either metabolic activity or the capacity to resume growth after intracellular acidification is yet undetermined and should be addressed in subsequent studies.

### 3.3 PKA and pH_c_ interaction: feedback loops upon nutritional transitions

Active protein kinase A is well known to promote fermentable growth and limit starvation survival. We now describe the role of PKA controlling pH_c_ *specifically* upon glucose depletion. It appears, from literature (Aoh et al., 2013) and our current work, that PKA activity has different effects upon glucose addition than at glucose depletion. This may seem remarkable, but is much less so in the light of the kinase targets present. Upon both glucose starvation or addition, global changes of protein expression occur (Boy‐Marcotte et al., 1996; Radonjic et al., 2005) and both catalytic and regulatory subunits of PKA itself change localisation (Tudisca et al., 2010). The completely different set of targets dictates that the role of PKA during glucose depletion is not simply the reversal of that during addition.

This is one of the reasons why comparison of mutants may give ambiguous results, because here too the history is different, and therefore, the network present is not the same. The only way to study such densely feedbacked networks is through carefully times interventions and time‐resolved analysis thereof.

Cytosolic pH has been suggested to set growth rate at least partially via inhibition of the PKA pathway (Dechant et al., 2014; Orij et al., 2012). By analysing the effects of PKA on pH_c_, we identified a new role for pH_c_ in a nutritional transition. In the presence of glucose, pH_c_ limits growth rate, while in its absence it limits survival, defined as the number of cells able to form colonies after 48 hr under optimal growth conditions. Therefore, upon glucose addition a pH_c_ decrease contributes to the *activation* of PKA (Colombo et al., 1998; Thevelein & De Winde, 1999), during growth, acidification gradually reduces growth rate possibly through PKA (Dechant et al., 2010, 2014), whereas upon glucose depletion it is PKA activity that causes most of the pH_c_ decrease. Our work establishes the mutual interaction between PKA and pH_c_ as a major control node of nutritional transitions.

## 4 EXPERIMENTAL PROCEDURES

### 4.1 Yeast strains and plasmids

Strains used in this work are listed in Supporting information Table S1. Unless stated otherwise, all strains are derivatives of the wild‐type laboratory strain BY4741. The strain *pde1Δpde2Δ *was obtained by PCR‐based deletion of *PDE2* in the *pde1Δ* strain from the yeast knockout collection (EUROSCARF). The strain *ira1Δira2Δ* was generated by integrating the partial deletion cassette of *IRA1* amplified from the strain PM903 (a kind gift from Dr. JM Thevelein; Colombo et al., 1998) in the strain *ira2Δ* (EUROSCARF). The *TPK1^as^tpk2∆tpk3∆* was obtained by successive deletion of *TPK2* and *TPK3* in BY4741 and the replacement of TPK1 with the *TPK1^M164G^*. We first integrated the *BleMX4* marker in front of the *TPK1* promoter in the strain Y3561 (a kind gift from Dr. JR Broach; Zaman et al., 2009) which already contained the *TPK1^M164G^*point mutation. Then, we amplified the region spanning from the marker to the *TPK1^M164G^*mutation and transformed it into our strain. Proper integration of the selection markers was checked by PCR, and sequencing was used to verify the introduction of the *TPK1^M164G ^*allele.

*PDE2* overexpression (BY+*PDE2 *o.e.) was achieved by introducing plasmid pM387 (a kind gift from Dr. PK Herman). BY4741 transformed with an empty vector (pYES2; BY4741+E.V.) was used as a control in experiments with this strain.

Plasmids used in this work are listed in Table S2. For sub‐cloning of pHluorin into a high copy vector with a *HIS3* selection marker, we introduced the *Xho*I‐*Kpn*I fragment of the pKS1 plasmid (Dualsystems Biotech; Switzerland) containing the *CYC1* terminator into the pRSII323 vector (Addgene plasmid 35463) digested with the same enzymes. The resulting vector was digested with *Spe*I a *Pst*I and the 1.2 kbp *Spe*I‐*Pst*I fragment of pYES2‐*P_ACT1_*‐pHluorin containing both the *ACT1* promoter and the pHluorin gene was inserted, generating the new pHluorin vector pRSII323‐*P_ACT1_*‐pHluorin. The proper integration of the inserts was verified by restriction analysis after each cloning step and the pHluorin gene was sequenced in the final plasmids. The sequencing data reported a point mutation in the *ACT1* promoter (A‐>C, position −346 from pHluorin ATG), but this did not have negative effects on expression.

Primers used for strain generation and plasmid verification can be found in Supporting information Table S3.

### 4.2 Culture conditions and treatments

Strains were maintained in Synthetic Complete medium. This medium contained 1.7 g/L of yeast nitrogen base (YNB) without amino acids and without ammonium sulphate, 1 g/L of sodium glutamate monohydrate, 20 g/L of glucose and the appropriate Kaiser dropout mix supplement (ForMedium, Norfolk, UK). For the pH_c_ measurements, cultures were pre‐grown and maintained in low fluorescence medium which contained YNB without amino acids, without ammonium sulphate without folic acid without riboflavin (MP biomedicals, Huissen, The Netherlands) instead of the aforementioned standard YNB. All media were buffered at pH 5.0 with 25 mM sodium citrate, unless indicated otherwise. The OD_600_ indicated as a starting point of the experiments were measured in a Lightwave II table spectrophotometer (Isogen life sciences, The Netherlands).

Cyclic adenosine monophosphate (cAMP) (Sigma‐Aldrich; St. Louis, MO, USA) was added at the indicated concentrations from a 200 mM stock in water adjusted to pH 5 with NaOH. 1NM‐PP1 (4‐Amino‐1‐tert‐butyl‐3‐(1ʹ‐naphthylmethyl)pyrazolo[3,4‐d]pyrimidine; Calbiochem, EMD Millipore, Billerica, MA USA) was used from 2 mM stocks in DMSO. Ebselen (2‐phenyl‐1,2‐benzisoselenazol‐3(2H)‐one; AG scientific Inc, San Diego, CA, USA) was added at 10 µM from DMSO stocks at 10 mM. All stocks were stored at −20°C.

### 4.3 Cytosolic pH measurements

Cytosolic pH was monitored using the GFP‐derivative ratiometric pHluorin expressed from the plasmid pYES2‐*P_ACT1_*‐pHluorin or pRSII323‐*P_ACT1_*‐pHluorin. Essentially, pH_c_ measurements were performed as described previously (Orij et al., 2012): Strains expressing pHluorin were transferred to microtitre plates. Fluorescence emission at 510 nm was registered upon excitation at 390 and 470 nm, along with OD_600_ in a FLUOstar OPTIMA microplate reader (BMG Labtech, Germany). Background fluorescence from the media and culture autofluorescence were subtracted for each wavelength, and the ratio 390 nm/470 nm was calculated. Culture autofluorescence was calculated from the OD_600_ vales from a calibration curve previously obtained for untransformed BY4741. The 390 nm/470 nm ratio was transformed into pH_c_ values using a calibration curve, which was obtained by measuring the background‐corrected ratio 390/470 in BY4741 expressing pYES2‐*P_ACT1_*‐pHluorin permeabilised with digitonin and resuspended in buffers covering a pH range from 5 to 8.

### 4.4 Growth curves

In growth curve experiments, yeast cultures were pre‐grown overnight in Synthetic Complete medium in glass tubes until glucose depletion and diluted to OD_600_ 2 (~1:10 dilution) in low fluorescence medium in microtitre plates. Growth (OD_600_) and pH_c_ were then monitored for 18–30 hr every 10 min. Fluorescence microscopy inspection was used to check that the pHluorin signal remained cytosolic at the end of the growth curves.

To better compare the pH_c_ profiles for the set of PKA mutants analysed, we summarised the information of the pH_c_ profiles by calculating the pH_c_ during growth and the pH_c_ after glucose depletion. For such calculations, we took into account that the different strains presented different lag phases and/or growth rates and therefore depleted glucose at different times. We defined the pH_c_ during growth as the average pH_c_ during the hour after the population had undergone two OD_600_ doublings. Cytosolic pH after glucose depletion was defined as the average pH_c_ measured between 10 to 11 hr after the moment of glucose depletion, set as the time‐specific growth rate (µ) decreased below 0.02 per hr. Growth rates were calculated as reported previously (Orij et al., 2012).

### 4.5 Starvation experiments

For starvation experiments, yeast cultures were grown overnight to exponential phase (OD_600_~5–10, ~0.25–0.5 of maximal OD_600_) in Erlenmeyer flasks in low fluorescence medium. Pre‐treatments were also performed in flasks to ensure proper aeration of the cultures. Cultures were aliquoted in 1.5 ml tubes, washed twice with fresh low fluorescence medium without glucose, resuspended in this medium and transferred to microtitre plates. Cytosolic pH was then monitored for 1 hr every 5 min. Note that because of the washing and preparation time, pH_c_ is not measured immediately after the starvation and the first minutes of the pH_c_ drop are not determined.

In Figure 4b–g, cultures exponentially growing overnight on different starting concentrations of glucose were diluted in fresh medium with the same concentration of glucose and grown for at least one doubling before the experiment. In that way, we ensured that the cultures were not near glucose depletion at the moment of the experiment.

### 4.6 Starvation at different external pH (pH_ex_) and viability assay

To assess viability after glucose starvation, we followed the starvation protocol detailed above with the following variations. Starvation media were adjusted to various pH (pH_ex_) by adding buffers except for pH_ex_ 3, for which pH was set with HCl. Tartaric acid 25 mM was used to buffer at pH 4, 25 mM sodium citrate for pH 5, 50 mM MES for pH 6 and 100 mM MOPS for pH 7. The actual pH of the media after buffer addition was as follows: 4.4 for pH_ex_ 4, 5.3 for pH_ex_ 5, 6.3 for pH_ex_ 6. For pH_ex_ 7, two different buffer stocks at pH 7 and 6.8 were used, leading to small differences in viability among the experiments (Figure 5e vs. f). After the washes, the starved cultures were transferred to glass tubes. Tubes were subsequently incubated in a rotating wheel at 30°C. Samples were taken to monitor pH_c _and assess viability. To evaluate survival, samples were serially diluted and plated on YPD immediately after the transfer to starvation conditions and three days later. Plates were incubated at 30°C for about 48 hr, and the number of colony‐forming units was determined. The number of colony‐forming units immediately after the transfer to starvation conditions was considered as the reference point (100%) to determine the percentage of viable cells at day 3.

### 4.7 Statistical analysis

Biological replicates were performed on different days. Statistical analysis was performed with GraphPad Prism 6 software. Unless otherwise indicated, significance between conditions was evaluated by using ANOVA with Bonferroni's multiple comparison correction of the p‐values. Gaussian distribution of the data was assumed.

Biological variability was high in starvation experiments and affected the absolute pH_c_ values but not the differences between pH_c_ under different treatments. This suggests that additional factors yet to be identified affect absolute pH_c_ under these conditions. To exclude these confounding factors from our statistical analysis, we performed paired comparisons in these set of data (Figures 3 and 4c–g).

In the figures, significance is indicated as follows: n.s., not significant; *p‐value ≤ 0.05.

## CONFLICT OF INTEREST

None Declared.

## AUTHOR CONTRIBUTIONS

LDE and GJS conceived of the experiments; LDE and MvdD performed the experiments; LDE and GJS performed the data analysis and interpretation; LDE, GJS and SB wrote the manuscript.
