# Supplementary material for: Caloric restriction controls stationary phase survival through Protein Kinase A (PKA) and cytosolic pH
Source: Aging Cell. 2019 Feb 20;18(3):e12921. doi: 10.1111/acel.12921 (PMC6516148; doi:10.1111/acel.12921)
Supplement: Supplementary file 1 [file ACEL-18-e12921-s001.docx]

Supporting information

**Table S1**. Strains used in this work.

| Strain name | Genotype | Strain code and reference |
| --- | --- | --- |
| BY4741 | *MATa his3Δ1 leu2Δ0 met15Δ0 ura3Δ0* | EUROSCARF |
| *bcy1Δ* | BY4741 *bcy1::kanMX4* | MAR231 (Casado *et al.*, 2011) |
| *pde1Δpde2Δ*, | BY4741 *pde1Δ::KanMX4 pde2Δ::BleMX4* | LDE042 this work |
| *ira1Δira2Δ*, | BY4741 *ira2Δ::KanMX4 ira1::LEU2* | LDE043, this work |
| DAmP *CYR1* | *MATa his3Δ1 leu2Δ0 met15Δ0 ura3Δ0* *CYR1-kanR;* BY4741-derived | Yeast Decreased Abundance by mRNA Perturbation (DAmP) collection |
| *TPK1^as^tpk2∆tpk3∆* | BY4741 *tpk1*::*BleMX4-P_TPK1_-TPK1^M164G^ tpk2∆::NatMX4 tpk3∆::KanMX4* | LDE147, this work |
| M5 | *MATα* *leu2-112 ura3-52 trp1-92*  (M5 segregant) | YSH6.36-1C; (Versele, *et al*., 2004) |
| *fil1* | M5 *fil1* (CYR1-E1682K) | PVD1150, (van Dijck *et al*., 2000; Versele *et al*., 2004) |
| *TPK1^as^tpk2∆tpk3∆* (W303-background) | W303-1B *Mat a ade2–1 can1–100 his3–11,15 leu2–3,112 trp1–1 ura3–1 tpk2::KAN tpk3::TRP1 tpk1^M164G^* | Y3175, M.C. Duncan *(Stephan, et al*., 2009) |

**Table S2**. Plasmids used in this work.

| Plasmid name | Description | Plasmid code and reference |
| --- | --- | --- |
| pYES2 | 2µ, *Amp^R^*, *URA3* |  |
| pRSII323 | 2µ, *Amp^R^*, *HIS3* | Addgene plasmid #35463 (Chee & Haase, 2012) |
| pRS40N | *Amp^R^*, *bleMX4* | Addgene plasmid #35480 (Chee & Haase, 2012) |
| pRS40B | *Amp^R^*, *natMX4* | Addgene plasmid #35478 (Chee & Haase, 2012) |
| pKS | 2µ, *Amp^R^*, *P_ADH2_*-*T_CYC1_* | Dualsystems Biotech, Switzerland |
| pYES2-*P_ACT1_*-pHluorin | pYES2; *P_ACT1_*-pHluorin | (Orij *et al*., 2009) |
| pRSII323-*P_ACT1_*-pHluorin | pRSII323; *P_ACT1_*-pHluorin | This work |
| *PDE2* o.e | high copy, *Amp*, *URA3* | pM387 or pPHY1107; (Ramachandran & Herman, 2011) |

**Table S3**. Primers used in this work. Forward and Reverse plasmids are indicated as F or R, respectively. In gene replacement primers, the underlined sequences indicate the plasmid region used to amplify the antibiotic-resistance marker.

| Primer name | SEQUENCE | Description |
| --- | --- | --- |
| pLDE016 | 5’-CATTCTTTTTGAGATCACTACTACTTA ATTGAAGAAAACATAACCTATTGGGGGCTGGCTTAACTATG-3’ | *pde2Δ*::*BleMX4* amplification from plasmid pRS40B (F) |
| pLDE017 | 5’-ATATTTATATGTTTATACAATGAATGG TACAAGAAATTTTGATATTCTTGTCTCCTTACGCATCTGTG-3’ | *pde2Δ*::*BleMX4* amplification from plasmid pRS40B (R) |
| pLDE020 | 5’-TTTGGATTCAATGGACGTG-3’ | *ira1Δ*::*LEU2* amplification from strain PM903 (F) |
| pLDE021 | 5’-GAAAACGAATGACCCAACG-3’ | *ira1Δ*::*LEU2* amplification from strain PM903 (R) |
| pLDE032 | 5’-CAAATATACAGCCGGCACAAACA GCAGCTTCACTCAGGTTAACTCA GGGGCTGGCTTAACTATG-3’ | *tpk2Δ*::*NatMX4* amplification from plasmid pRS40N (F) |
| pLDE033 | 5’-TTGGTGGAGGAAAAAAGAGAAA GGAAAAGGAGTGAGAGAAAGTAC TCTCCTTACGCATCTGTG-3’ | *tpk2Δ*::*NatMX4* amplification from plasmid pRS40N (R) |
| pLDE068 | 5’-TGACATTCTTCCTAAGAATTTACAA GAACCCTCCGCTGGACGGGCCTTAA GGGGCTGGCTTAACTATG-3’ | *P_TPK1_* primer. Amplify *BleMX4* integrative cassette from plasmid pRS40B (F) |
| pLDE069 | 5’-CTTCGAACAAGTAACTAAAAATATGT GGTGTATACGGTATGTAAATGTTT TCTCCTTACGCATCTGTG-3’ | *P_TPK1_* primer. Amplify *BleMX4* integrative cassette from plasmid pRS40B (R) |
| pLDE078 | 5’-ATATTGTATATCGGTGGTTGTACAA GGAAAGAGCGAGCCTGCACAAAATG GGGGCTGGCTTAACTATG-3’ | *tpk3Δ:: KanMX4* amplification from plasmid pKS (F) |
| pLDE079 | 5’-ATTGATTTTTTTTTTTTTTCAATTAC AATTATCCCACTGAACCTCCTTAA TCTCCTTACGCATCTGTG-3’ | *tpk3Δ:: KanMX4* amplification from plasmid pKS (R) |
| pLDE018 | 5’-TGCGTCCTTTTCTAGTTGC-3’ | *PDE2* primer; verification of *pde2Δ::BleMX4* (F) |
| pLDE014 | 5’-CTATGAAAGGTTGGGCTTC-3’ | *BleMX4* primer. Deletion cassette integration verification (R) |
| pLDE022 | 5’-TTACGACGGAGAGGTTGAC-3’ | *IRA1* primer; verification of *ira1::LEU2* (F) |
| pLDE023 | 5’-ATTGGTGTCGGATCTTCTC-3’ | *IRA1* primer; verification of *ira1::LEU2* (R) |
| pLDE005 | 5’-AACAATACACCGTTCCAG-3’ | *LEU2* internal verification of *ira1::LEU2* (R) |
| pLDE072 | 5’-AAGGAAATTAAGGGGGAG-3’ | *TPK2* primer; *tpk2Δ::NatMX4* verification (F) |
| pLDE073 | 5’-TATAAAGAAAGTGCGCCAG-3’ | *TPK2* primer; *tpk2Δ::NatMX4* verification (R) |
| pLDE015 | 5’-GATTTGCCACTGAGGTTC-3’ | Antibiotic cassette (*NatMX4/BleMX4*) promoter primer. Deletion cassette integration verification (R) |
| pLDE071 | 5’-CCCATCCTCCTTAAGCTA-3’ | Primer upstream *P_TPK1_*. Verification of *BleMX4* integration in front of *P_TPK1_* (F) |
| pLDE050 | 5’-GTGCTATGTAGTCGGGAG-3’ | *TPK1* primer. Verification of *BleMX4* integration in front of *P_TPK1_* (PCR and sequencing; R). |
| pLDE070 | 5’-GGGGTGGTTTTTGACATC-3’ | *TPK1* primer. *BleMX4-P_TPK1_-TPK1^M164G^* integration verification (PCR and sequencing; R) |
| pLDE083 | 5’-GAACCTCAGTGGCAAATC-3’ | Antibiotic cassette (*NatMX4/BleMX4*) promoter primer. *BleMX4-P_TPK1_-TPK1^M164G^* integration verification (F) |
| pLDE065 | 5’-CCAAGAAAAACCGAAGCA-3’ | *TPK1* primer. *TPK1^M164G^* verification (PCR and sequencing; F) |
| pLDE066 | 5’-TCCACCTTCAATATAATCCCC-3’ | *TPK1^M164G^* allele-specific primer. *TPK1^M164G^* verification (R) |
| pLDE076 | 5’-GGTGAACCACTTTCTTTTTAGTGAA-3’ | *TPK3* primer; *tpk3Δ::KanMX4* verification (F) |
| pLDE077 | 5’-TCTTCTTATTGTAGCAGGCTCACTT-3’ | *TPK3* primer; *tpk3Δ::KanMX4* verification (R) |
| pLDE029 | 5’-CCGATTTAGAGCTTGACG-3’ | *pHluorin sequencing primer (F)* |
| pLDE028 | 5’-CGTGAATGTAAGCGTGAC-3’ | *pHluorin sequencing primer (R)* |
| pLDE061 | 5’-GGGATCTGGTAAAAGTTTG-3’ | *TPK1as sequencing primer (F)* |


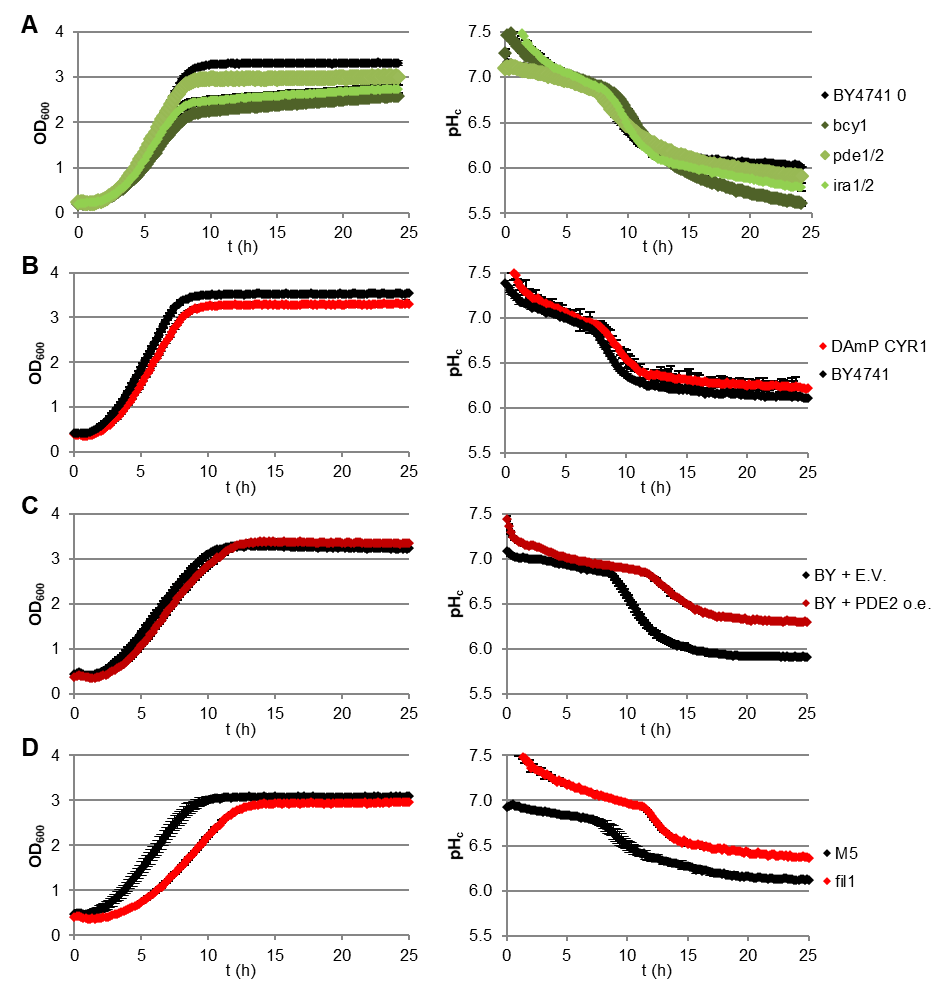


**Figure S1**. A representative growth (left panel) and pH_c_ (right panel) curve for each of the PKA mutants analysed in Fig 1 (see legend on the right).

(A) Overactive PKA mutants (green) derived from the parental BY4741 (black) (B-D) Low PKA activity mutants (red) and their respective parental strains BY4741 (B), BY4741+E.V. (C) and M5 (D) Error bars represent SD of three technical repeats.

**
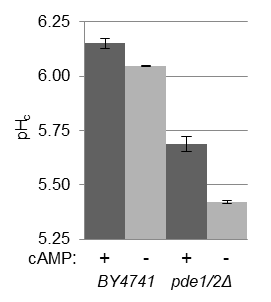
**

**Figure S2**. The effects of cAMP on pHc are exacerbated in a strain lacking the phosphodiesterases (*pde1Δpde2Δ*).

20 mM cAMP (+) or water (-) were added to growing cultures at 4h after inoculation as in Fig 2. The pH_c_ at the end of the growth curve (16 h after inoculation) is shown. Data are averages ± SD of three biological replicates.

**
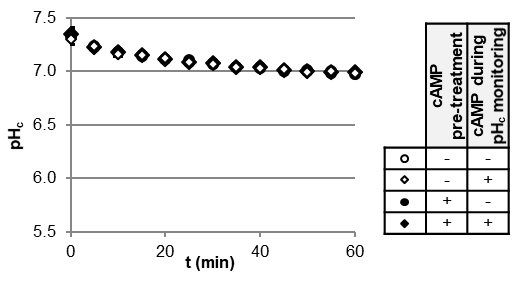
**

**Figure S3**. Addition of cAMP does not affect pH_c_ in the presence of glucose.

Exponentially growing BY4741 cultures were subjected to a 90 min pre-treatment with or without cAMP as in Figure 3 and then washed and resuspended in fresh media containing glucose in the presence or absence of cAMP. Data shown are averages of two biological replicates ±SD.

**
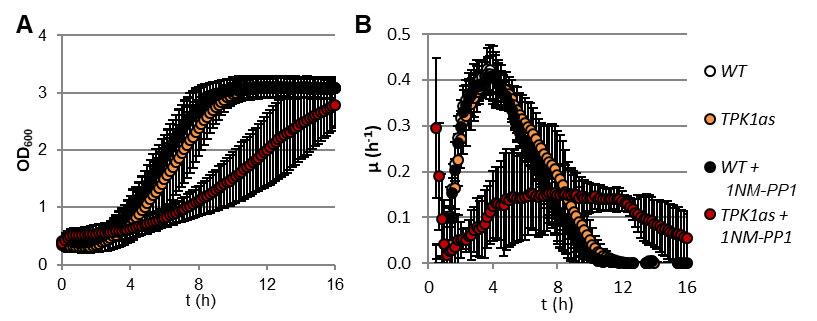
**

**Figure S4**. Addition of 1NM-PP1 strongly inhibits growth rate in *TPK1^as^tpk2∆tpk3∆* but not in the parental strain BY4741.

A growth experiment of the indicated strain was performed in the presence of 1NM-PP1 or drug vehicle (DMSO). The OD_600_ (A) and the growth rate (µ; B) profile over time are shown. Data shown are averages ±SD of four biological replicates.

**
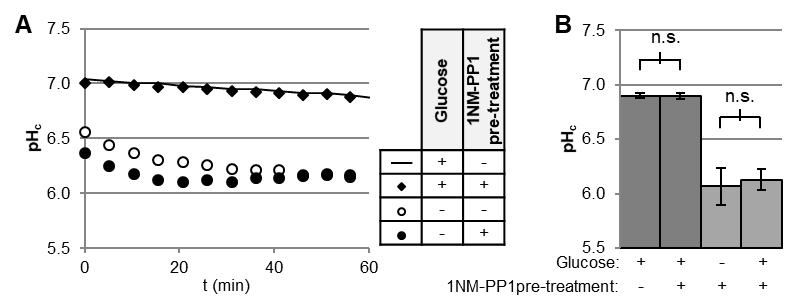
**

**Figure S5**. Pre-treatment with 1NM-PP1 does not interfere with pH_c_ regulation in BY4741.

(A) A representative result of the pH_c_ profile of BY4741 treated as in Fig 3C. (B) pH_c_ 60 minutes after starvation for the indicated treatments. Data shown are averages ±SD of three biological replicates.

**
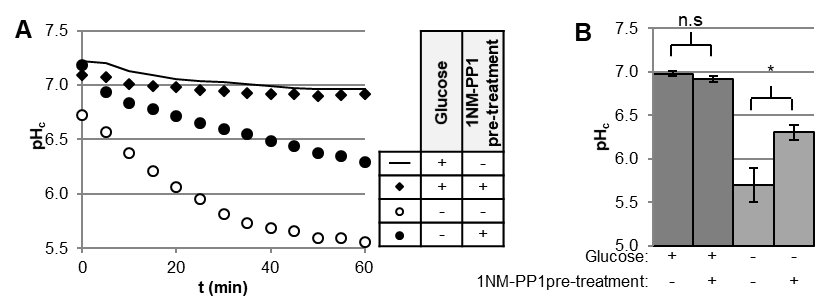
**

**Figure S6**. PKA inhibition in *TPK1^as^tpk2*∆*tpk3*∆ (W303-background) strain also prevents cytosolic acidification upon glucose depletion.

(A) Exponentially growing *TPK1asTPK2asTPK3as* were treated as in Fig 3C. A representative result of pH_c_ dynamics is shown. (B) pH_c_ 60 minutes after starvation for the indicated treatments as in (A). Data shown are averages ±SD of three biological replicates.


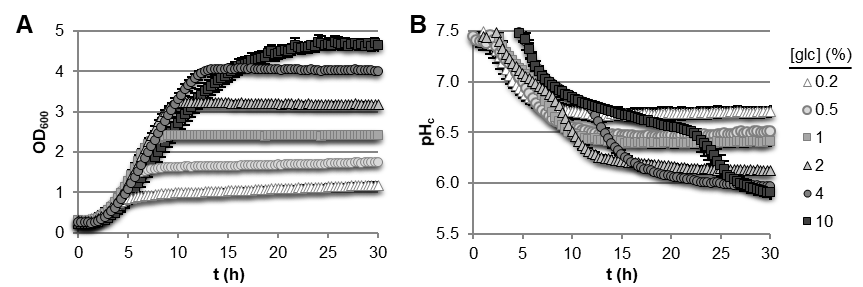


**Figure S7**. Glucose abundance regulates pH_c_ upon glucose depletion.

A representative growth (OD_600_; A) and pH_c_ (B) curve over time for the parental strain BY4741 growing on media with the indicated initial concentrations of glucose.


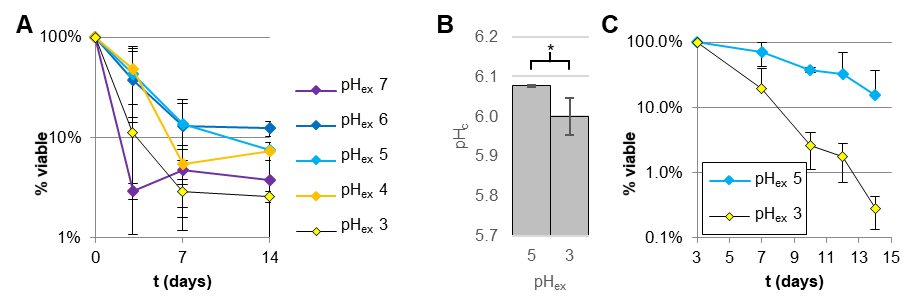


**Figure S8**. Effects of pH_ex_ changes on long term starvation survival (A) and CLS (B). (A) Cultures of BY4741 were starved for glucose at different pH_ex_ and as in Fig 5 and viability was determined at the indicated times. Data shown are averages ±SD of two biological replicates. (B-C) Cytosolic pH after natural glucose depletion in BY4741 cultures grown at the indicated pH_ex_ (B) and chronological lifespan of the same cultures monitored as CFU counts relative to day 3. Data shown are averages ±SD of two biological replicates.

.**
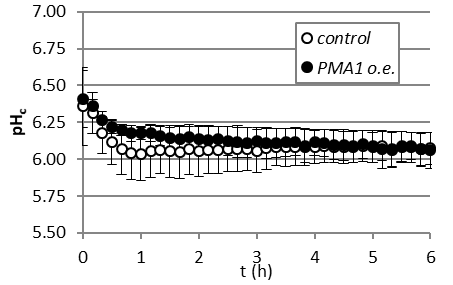
**

**Figure S9**. Overexpression of PMA1 does not increase pH_c_ upon glucose starvation. Cytosolic pH was monitored after glucose starvation of glucose growing cultures of BY4741 transformed with the integrative vectors pAG306-Gal-Pma1 (Henderson *et al.*, 2014) and pATC1-GEV (Veatch *et al.*, 2009). Cultures were treated with 5 µM of estradiol (PMA1 o.e.) or vehicle (control) for 2 h prior starvation. Estradiol addition to this strain activates the GEV (Gal4-EBD-VP16) transcription factor which specifically drives the expression of GAL1 promoters, including that of the extra integrated copy of *PMA1* (Henderson *et al.*, 2014). Data represent averages ±SD of two biological repeats

**
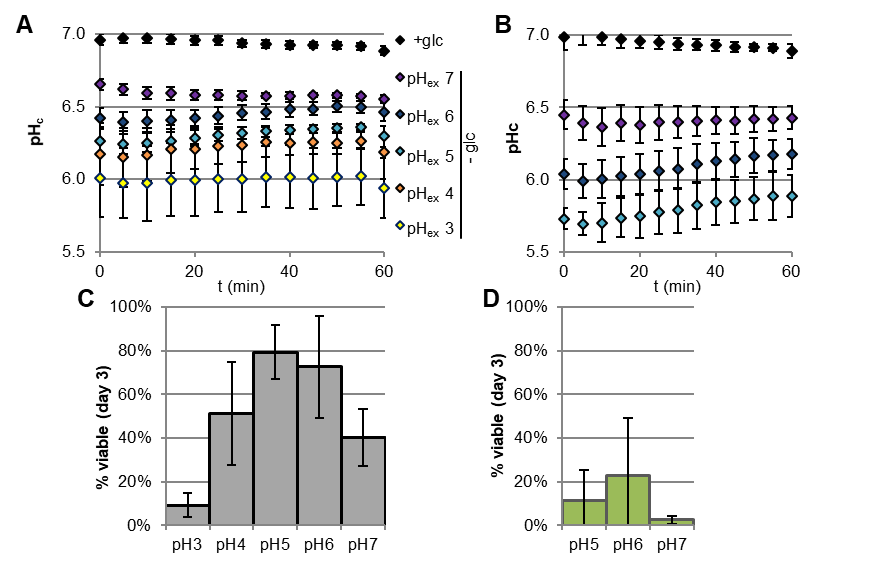
**

**Figure S10**. Increasing starvation pH_c_ does not restore the reduced starvation survival of a high PKA mutants.

Cytosolic pH dynamics upon glucose depletion for BY4741 (A) and *ira1/2Δ* (B). (C-D) % of viable cells after three days of glucose starvation in the conditions shown in (A) and (B), respectively. Data shown represent the averages (±SD) of at least three biological replicates.

References

Casado, C., González, A., Platara, M., Ruiz, A., & Ariño, J. (2011). The role of the protein kinase A pathway in the response to alkaline pH stress in yeast. *The Biochemical Journal*, *438*(3), 523–533. https://doi.org/10.1042/BJ20110607

Chee, M. K., & Haase, S. B. (2012). New and Redesigned pRS Plasmid Shuttle Vectors for Genetic Manipulation of Saccharomyces cerevisiae. *G3; Genes|Genomes|Genetics*, *2*(5), 515–526. https://doi.org/10.1534/g3.111.001917

Henderson, K. a, Hughes, A. L., & Gottschling, D. E. (2014). Mother-daughter asymmetry of pH underlies aging and rejuvenation in yeast. *ELife*, *3*, e03504. https://doi.org/10.7554/eLife.03504

Orij, R., Postmus, J., Beek, A. T., Brul, S., Smits, G. J. G. J., Ter Beek, A., … Smits, G. J. G. J. (2009). In vivo measurement of cytosolic and mitochondrial pH using a pH-sensitive GFP derivative in Saccharomyces cerevisiae reveals a relation between intracellular pH and growth. *Microbiology*, *155*(1), 268–278. https://doi.org/10.1099/mic.0.022038-0

Ramachandran, V., & Herman, P. K. (2011). Antagonistic interactions between the cAMP-dependent protein kinase and tor signaling pathways modulate cell growth in Saccharomyces cerevisiae. *Genetics*, *187*(2), 441–454. https://doi.org/10.1534/genetics.110.123372

Stephan, J. S., Yeh, Y.-Y., Ramachandran, V., Deminoff, S. J., & Herman, P. K. (2009). The Tor and PKA signaling pathways independently target the Atg1/Atg13 protein kinase complex to control autophagy. *Proceedings of the National Academy of Sciences of the United States of America*, *106*(40), 17049–17054. https://doi.org/10.1073/pnas.0903316106

van Dijck, P., Ma, P., Versele, M., Gorwa, M.-F. F., Colombo, S., Lemaire, K., … Loïez, A. (2000). Baker’s Yeast Mutant (fil 1) With a Specific, Partially Inactivating Mutation in Adenylate Cyclase Maintains a High Stress Resistance During Active Fermentation. *Journal of Molecular Microbiology and Biotechnology*, *2*(4), 521–530. Retrieved from http://www.ncbi.nlm.nih.gov/pubmed/11075928%5Cnhttp://www.horizonpress.com/jmmb/v2/v2n4/27.pdf

Veatch, J. R., McMurray, M. A., Nelson, Z. W., & Gottschling, D. E. (2009). Mitochondrial Dysfunction Leads to Nuclear Genome Instability via an Iron-Sulfur Cluster Defect. *Cell*, *137*(7), 1247–1258. https://doi.org/10.1016/J.CELL.2009.04.014

Versele, M., Thevelein, J. M., & Van Dijck, P. (2004). The high general stress resistance of the Saccharomyces cerevisiae fill adenylate cyclase mutant (CyrlLys1682) is only partially dependent on trehalose, Hsp 104 and overexpression of Msn2/4-regulated genes. *Yeast*, *21*(1), 75–86. https://doi.org/10.1002/yea.1065
